# Significant and Systematic Expression Differentiation in Long-Lived Yeast Strains

**DOI:** 10.1371/journal.pone.0001095

**Published:** 2007-10-31

**Authors:** Chao Cheng, Paola Fabrizio, Huanying Ge, Min Wei, Valter D. Longo, Lei M. Li

**Affiliations:** 1 Molecular and Computational Biology Program, Department of Biological Sciences, University of Southern California, Los Angeles, California, United States of America; 2 Andrus Gerontology Center and Department of Biological Sciences, University of Southern California, Los Angeles, California, United States of America; 3 Department of Mathematics, University of Southern California, Los Angeles, California, United States of America; University of Michigan, United States of America

## Abstract

**Background:**

Recent studies suggest that the regulation of longevity may be partially conserved in many eukaryotes ranging from yeast to mammals. The three yeast mutants *sch9*Δ, *ras2*Δ, *tor1*Δ show extended chronological life span up to three folds. Our aim is to dissect the mechanisms that lead to the yeast life span extension.

**Methodology/Principal Findings:**

We obtain gene expression profiles of *sch9*Δ, *ras2*Δ, *tor1*Δ as well as that for a wild type at day 2.5 in SDC medium using Affymetrix Yeast2.0 arrays. To accurately estimate the expression differentiation between the wild type and the long-lived mutants, we use sub-array normalization followed by a variant of the median-polishing summarization. The results are validated by the probe sets of *S. pombe* on the same chips. To translate the differentiation into changes of biological activities, we make statistical inference by integrating the expression profiles with biological gene subsets defined by Gene Ontology, KEGG pathways, and cellular localization of proteins. Other than subset-versus-other comparisons, we also make local comparisons between two directly-related gene subsets such as cytosolic and mitochondrial ribosomes. Our consensus is obtained by cross-examination of these inferences. The significant and systematic differentiation in the three long-lived strains includes: lower transcriptional activities; down-regulation of TCA cycle and oxidative phosphorylation versus up-regulation of the KEGG pathway Glycolysis/Gluconeogenesis; the overall reduction of mitochondrial activities. We also report some different expression patterns such as reduction of the activities relating to mitosis in *ras2*Δ.

**Conclusions/Significance:**

The modification of energy pathways and modification of compartment activities such as down-regulation of mitochondrial ribosome proteins versus up-regulation of cytosolic ribosome proteins are directly associated with the life span extension in yeast. The results provide a new and systematic *S. cerevisiae* version of the free radical theory from the perspective of functional genomics.

## Introduction

Recent findings suggest that ageing, like many other biological processes, is subject to regulation by pathways that may have been partially conserved throughout evolution [1], [2]. In fact, the down-regulation of the glucose-sensing/insulin/IGF-1 pathway promotes life span extension in organisms ranging from yeast to mice. In *S. cerevisiae* two different paradigms are used to measure longevity: the replicative life span, which is defined as the total number of daughter cells generated by a mother cell; and the chronological life span which is measured by monitoring the mean/maximum survival time of a population of non-dividing yeast. Here we focus on the chronological life span, which represents a simple but valuable system to study how post-mitotic cells age [3]. The mutations of a single gene within the principal yeast nutrient-sensing pathways can extend the chronological life span dramatically [4], [5]. Among these pathways, the Sch9, Ras2/cAMP/PKA and TOR pathways are of most interest. Importantly, in higher eukaryotes pathways that appear to share a common evolutionary origin with the Sch9 and TOR pathways are also implicated in life span regulation. In budding yeast inactivation of *sch9*, homolog of mammalian serine/threonine protein kinase Akt, extends chronological life span by nearly three folds [6]. Deletion of *ras2* or down-regulation of *cyr1* in the Ras2/cAMP/PKA pathway nearly doubles the chronological life span of yeast [6], [7]. In a large scale screening in yeast, several genes that encode components of the nutrient-responsive TOR pathway were found to increase the chronological life span [8].

A number of theories have been proposed to explain the mechanism of ageing. Among them are the disposable soma theory of ageing first suggested by Weismann and later developed by Kirkwood *et al.* [9], [10], [11], the accumulated mutation theory first proposed by Medawar in 1952 [12], the antagonistic pleiotropy theory proposed by Williams in 1957 [13], the programmed and altruistic ageing theory [14]. The free radical theory of ageing first proposed by Harman in the 1950s [15] is particularly relevant to the research reported in this article. According to this theory, ageing is a consequence of free radical damage. Later Harman extended the idea to implicate mitochondrial production of ROS in the 1970s [16].

The partial conservation of the life-span regulatory pathways suggests that they may have evolved in ancestral unicellular organisms in order to overcome periods of starvation. Calorie restriction (CR), which resembles the starvation conditions used to assess chronological life span, causes longevity extension in all the ageing model systems. In this article we analyze the gene expression profiles of chronologically long-lived yeast strains. Under this ageing paradigm, haploid yeast are grown in synthetic complete medium (SDC) until nutrients are depleted. Once yeast stop dividing, they are kept in the depleted medium. The viability of the cultures is monitored over time by measuring the colony forming units (CFUs) [3]. Incubation in nutrient-depleted SDC mimics the conditions normally encountered by yeast in the natural environment where microorganisms survive for long periods of time under starvation.

The modification of the chronological life span, is the end effect of genetic interventions such as the knockout of *sch9*, and environmental changes such as calorie restriction. Extensive results have been obtained in relating genotypes and the phenotype of life span. Our effort aims to understand the intermediate steps of the ageing mechanism.

The microarray technology allows us to measure the expression profiles of a living cell. We obtain the gene expression profiles of the long-lived mutants *sch9*Δ, *ras2*Δ, and *tor1*Δ together with a wild type at day 2.5 in SDC medium using Affymetrix yeast2.0 arrays. How to accurately estimate the differentiation between the wild type and the long-lived mutants is a key problem in our functional genomic study of ageing. Usually the estimation consists of two steps: normalization and summarization. Normalization aims to remove any non-biological difference generated in the reaction and read-out process while keep the real biological differentiation between a target and a reference sample. The invariant-set [17], [18] and quantiles [19], [20] normalization are two widely used methods in the literature. However, in our case, it is possible that the expression profiles of the long-lived mutants have substantial differentiation compared to that of the wild type. It is also possible that the differentiation is not symmetric. Thereby we adopt the sub-array normalization that is designed to preserve biological differentiation [21], [23].

How to translate the differentiation into changes of biological activities is another key problem in the functional genomics of ageing. Relatively complete bio- and genomic- databases exist of *S. cerevisiae*. They provide us with instruments for statistical inference. In this article, we infer significant modifications of biological activities by integrating the expression differentiation with three sources of biological knowledge: Gene Ontology (GO), KEGG pathways, and cellular localization of proteins. Our consensus inference is obtained by cross-examination of the inferences drawn from the three perspectives. Furthermore, to reduce the gap of statistical significance and biological significance, we compare the transcriptional activities of two “directly-related gene subsets” such as the first half and second half of an energetic pathway, or ribosome proteins in either mitochondria or cytosol. The idea of local inference follows the basic principles of statistical design proposed by Sir R. Fisher while the idea of consensus inference is one statistical view of systems biology.

In this work, we study the yeast ageing mechanism from the perspective of functional genomics. The three mutants, *sch9*Δ, *ras2*Δ, *tor1*Δ share the same phenotype: longer chronological life span. The significant and systematic expression differentiation underlying the phenotype can shed light on the mechanism of ageing. We show that the goal is achievable and from the current data set we identify some common and characteristic changes of biological activities, which may directly lead to longevity.

## Materials and Methods

### Sample preparation and Affymetrix GeneChip arrays

We obtained the gene expression profiles of yeast strains including wild type, *sch9*Δ, *ras2*Δ, *tor1*Δ cells at day 2.5. Specifically, all strains used were obtained from frozen stocks. Each strain was inoculated in 1 mL SDC and grown overnight. Saturated overnight cultures were then diluted into 3 flasks each containing 50 mL of culture. All samples were incubated at 30°C with shaking (2200 rpm) until day 2.5. Total RNA was isolated from day 2.5 post-diauxic yeast cultures (2.0×10^8^ cells) according to the acid phenol protocol. Briefly, yeast were collected by centrifugation, washed with cold water once, and resuspended in 400 µl of 10 mM Tris pH 7.5, 10 mM EDTA, 0.5% SDS. After adding 400 µl of warm acid phenol the cell suspension was incubated at 65°C for 20 minutes with vortexing every 5 minutes, centrifuged and the supernatant extracted twice with acid phenol and once with chloroform. Total RNA was recovered by precipitation with ethanol and cleaned up by using the RNAsy kit (Qiagen). RNA (5 µg/sample) was sent to the UCLA DNA array Core Facility. Total RNA from independent cultures of each strain was used as a template to synthesize complementary RNA (cRNA). The biotin-labeled cRNA was hybridized to Affymetrix GeneChip® Yeast2.0 Array. In sum, three biological replicates were obtained for each of wild type, *sch9*Δ, *ras2*Δ, and *tor1*Δ.

In the SDC medium, a substantial proportion of yeast cells are still dividing before day 2. At older ages, such as day 3–5, most of the cells become hypometabolic, which is associated with a dramatic drop in transcription. We harvest mRNA at day 2.5 so that we can extract enough mRNA for microarray experiment while avoid the noise introduced by the transcriptional activities of dividing cells.

### Normalization and summarization

After imaging process, the expression of each sample is represented by a CEL file, which includes the fluorescence intensities of all probes. Denote the three arrays of wild type, *sch9*Δ *ras2*Δ, and *tor1*Δ respectively by W1, W2, W3, S1, S2, S3, R1, R2, R3, T1, T2, T3. The conversion of probe intensities to expression values requires two statistical procedures: normalization and summarization. We applied the Sub-Sub normalization [21] to our data sets, aiming at giving enough protection to possible differentiation between the mutants and the wild type. The normalization is carried out in a pairwise fashion. Namely, for each wild type sample, three replicates of a mutant are normalized with respect to this reference. Take *sch9*Δ for example, the normalized arrays with respect to W1, W2, W3 are respectively denoted by S1\W1, S2\W1, S3\W1, S1\W2, S2\W2, S3\W2, S1\W3, S2\W3, S3\W3.

Our summarization is a modified version of the median polishing method [22] in RMA (the Bioconductor affy package http://www.bioconductor.org/). The median polishing summarization method is based on a two-factor model, which include the sample effect and probe-specific effect. Namely, the gene expression of each sample is estimated by adjusting each individual probe effect. In our situation, we group the wild type and normalized mutant arrays by the reference, and then summarize each group. Take *sch9*Δ for example. We summarize the four arrays (the reference plus three normalized) W1, S1\W1, S2\W1, S3\W1 together. This leads to three estimates of expression fold changes of the mutant versus the wild type. In total, we have nine estimates from three wild type references, and we take their median difference as the final estimate. Due to the nature of normalization [23], only the portion of differentiation that is not confounded with the reference array is estimable. In the above scheme, arrays in a summarization group correspond to the same reference. Thus we expect that the difference between a (normalized) mutant and a wild type is, for the most part, real differentiation. The median is a robust estimate that is consistent with the median polishing method. Our treatment of reference in normalization is somewhat different from existing methods, and correspondingly we use this group median polishing summarization to take into account the reference effect. We note that summarization is done at the probe set level. Roughly speaking, the modified median-polishing summarization aims to remove the reference-specific effect for each probe set as well as the probe-specific effect.

The Yeast2.0 Array contains probe sets for both *S. cerevisiae* and *S. pombe*. The observed fluorescence intensities of *S. pombe* probes are primarily due to cross hybridization, and we only use them in normalization. To some extent, they play the role of external controls. Most genes correspond to only one probe set in Yeast2.0 Array, and for genes with multiple probe sets we take the average of fold changes.

### Wilcoxon scoring of gene subsets

Based on the expression fold changes of the three mutant strains with respect to the wild type, we make inference about the modifications of biological activities using gene subsets defined by Gene Ontology (GO), KEGG Pathways, and cellular organelle (GFP fusion localization). A common theme of these analyses is as follows. Suppose we have *m* gene subsets *S*_1_,*S*_2_,…,*S_m_*. From the log ratios of expression levels of a mutant with respect to the wild type, we want to identify those subsets whose expressions are significantly up-regulated or down-regulated. Denote the union of these gene subsets by 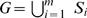. Our strategy consists of two steps. In the first step, for each subset *S_i_*, we compare their expressions against those in the complement of *S_i_* in *G* denoted by *G*−*S_i_*. This is a typical two-sample problem in statistics. We use the Wilcoxon rank test to calculate p-value (one-sided) for each comparison. In the second step, we rank these subsets according to their significances. These subsets could be a GO category, a metabolic pathway, or protein genes localized in an organelle. Other tests and methods such as GSEA [24] could be applied to our study. We report the results by the Wilcoxon scoring due to its well-established statistical properties such as robustness and reasonably good efficiency.

### Multiple test correction

To correct for multiple testing, we adjust the p-values by the method introduced by Storey *et al*. [25], [26]. In this method, the q-value is defined to evaluate the false discovery rate. The computation of q-values is implemented by the “qvalue” package provided in the R software (http://www.r-project.org/).

### Gene Ontology analysis

Gene ontology information is from “ftp://genome-ftp.stanford.edu/pub/go/”. The Gene Ontology subsets are defined from three related yet different aspects: biological processes, molecular functions and cellular components. Data structure for gene ontology (GO) is a directed acyclic graph (DAG). Each node in the DAG is a set of genes with specific annotation. The closer the nodes are to the terminal, the more detailed annotations are given and thereby are more informative. To avoid redundancy and overlapping between GO nodes and to facilitate our statistical analysis, we select from the DAG the nodes that are closest to the terminal and have at least 30 genes. This selection ends up with nodes of 44 cellular components, 53 molecular functions and 109 biological processes. The gene subsets defined by these nodes are referred to as terminal informative GO categories (TIGO). Then we apply the Wilcoxon scoring method and multiple test correction to these TIGO categories. By taking only the terminal informative TIGO categories rather than all the GO nodes, results are easier to be interpreted.

### KEGG Pathway analysis

The pathway information are from the KEGG database: http://www.genome.jp/kegg/. In total our study uses 103 *S. cerevisiae* pathways, most of which are well-established metabolic pathways. To expand our knowledge of ageing, we seek pathways that are significantly changed in the long-lived mutants. We regard each pathway as a subset of genes, and apply our statistical scoring and significance analysis to the 103 pathways and obtain a p-value and a q-value for each of them.

### Cellular organelle analysis

The cellular localization data are from http://yeastgfp.ucsf.edu/. In this data set, 75% proteins were classified into 22 distinct subcellular localization categories, including mitochondria, nucleus, nucleolus, vacuole, vacuole membrane, budding neck, etc. Many research indicate that mitochondrion plays a central role in ageing. We also expected that the cellular organelle analysis would provide some information about the role of the different organelles in ageing. In this analysis, genes that function in the same cellular localization are regarded as one gene subset. The protein gene subsets from the yeast GFP fusion localization database are different from the cellular components in the GO categories.

### Consensus and local Inference

**Figure 1:**
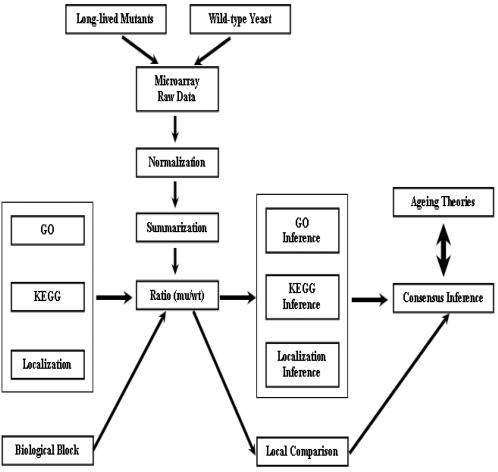
Scheme of consensus inference and local inference. After the preprocessing of the microarray data for the long-lived cells versus the wild type, we translate the differentiation into changes of biological activities by consensus and local inference using three kinds of biological instruments: Gene Ontology (GO), KEGG pathways, and cellular localization of proteins.

After making inference using each of the three biological instruments: Gene Ontology, KEGG Pathways, and cellular organelle, we can report a consensus by cross-examination. Our another approach is to compare two gene subsets in a natural “biological block”. For one example, we consider the first half and second half of an energetic pathway. For another example, we consider ribosomes in cytosol and ribosomes in mitochondria. Using this idea of local comparison, we get over the issue of multiple testing and thereby improve the statistical significance. The scheme of our inference is illustrated in Figure 1.

## Results

### Preprocessing of microarray data

We obtained the gene expression profiles of yeast strains including wild type, *sch9*Δ, *ras2*Δ, and *tor1*Δ cells at day 2.5 using Affymetrix GeneChip® Yeast2.0 Array. In total, three biological replicates were generated for each strain. RNA of these replicates was obtained from independent populations which were grown in separate flasks under similar conditions. The expression fold changes of 5841 yeast genes were obtained for *sch9*Δ, *ras2*Δ, and *tor1*Δ with respect to the wild type by the Sub-Sub normalization [21] followed by the modified median-polishing summarization (Materials and methods) that aims to remove the reference-specific effect. We optimized the parameters in the Sub-Sub normalization by examining the results among replicates, and by checking probe sets of *S. pombe* on the Yeast2.0 Array. In this normalization, we divide each array into sub-arrays and normalize probe intensities within each sub-array by least trimmed squares to protect differentiation. The subarray size is selected to be 50 by 50; subarrays overlap by half the subarray size; and the trimming fraction of least trimmed squares is 0.45.

**Figure 2:**
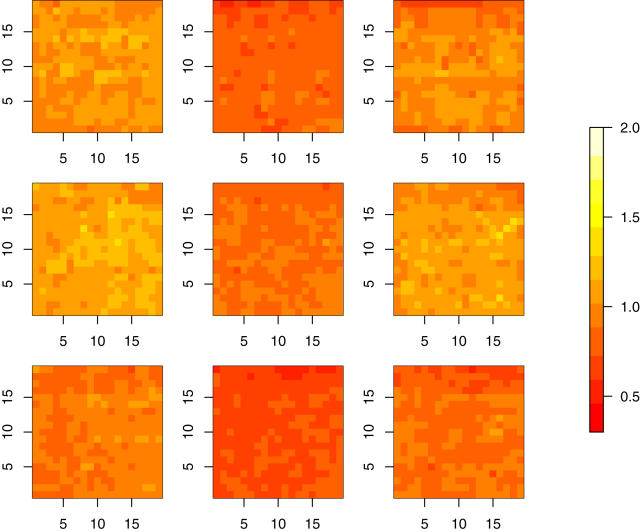
Non-homogeneous spatial patterns of relative scales. The estimates of the normalization scale in each subarray are shown by their spatial locations. We normalize each of the three target (sch9 mutant) arrays versus each of the three reference (wild type) arrays, and total nine spatial patterns are shown. Each row corresponds to a reference and each column corresponds to a target.

If the experiment conditions and mRNA amount for the reference and target samples are similar, we argue in [23] that a simple linear function is a good approximation in normalization even though the relationship between dye concentration and fluorescent intensity is nonlinear. Namely, to normalize a target array with respect to a reference, we shift and scale the probe intensities by α+β*target intensity in such a way that the differences with reference intensities are minimized. Specifically, the parameter α and β are estimated by least trimmed squares. In Figure 2, we show the estimates of the relative scale β in each subarray for *sch9*Δ versus wild type. Since we normalize each of the three target (mutant) arrays versus each of the three reference (wild type) arrays, total nine spatial patterns are shown in Figure 2, in which each row corresponds to a reference and each column corresponds to a target. The corresponding histograms of the scale parameters are shown in Figure S1. The adjustment of spatial effect can help reduce the variation between replicates, see examples in [21], [23]. In this case, for each gene the standard deviation of the nine (three targets versus three references, see Materials and methods) expression log-ratios of a mutant versus wild type is calculated. The medians of these standard deviations for *sch9*Δ, *ras2*Δ, *tor1*Δ are respectively 0.146, 0.125, 0.134.

**Figure 3:**
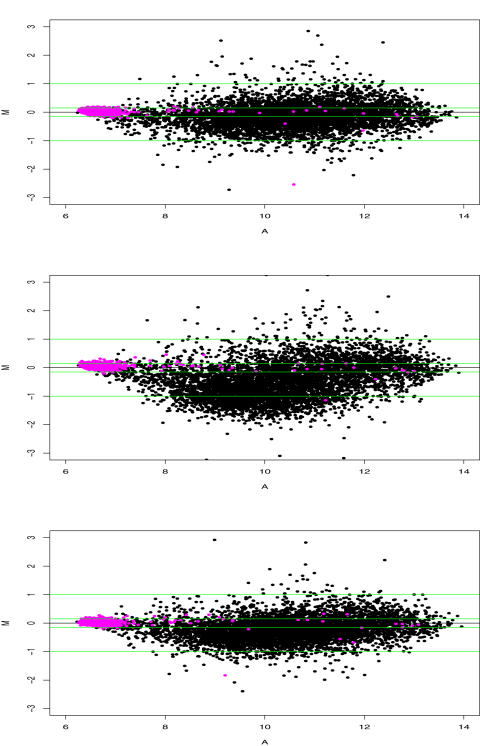
M-A plots of sub-sub normalization followed by modified median polishing summarization. In the M-A plots, the x, y coordinate value of a dot respectively show the average and difference of a gene expression between the wild type and a mutant. The differentiation for most probe sets of S. pombe are around zero as expected. Black: S. cerevisiae; Magenta: S. pombe. Top: sch9 mutant; Middle: ras2 mutant; Bottom: tor1 mutant. Two horizontal lines at ±0.15 are also plotted.

In Figure 3 we show the M-A plots of expression levels of three mutants versus the wild type. In the M-A plots, the x, y coordinate value of a dot respectively show the average and difference of a gene expression levels between a mutant and the wild type. The differentiation for probe sets of *S. pombe* are around zero as expected, especially at the left end. In fact, the medians of the average log ratios for *S. pombe* genes are 0.018, 0.047, 0.031 respectively for *sch9*Δ, *ras2*Δ, *tor1*Δ. Some probe sets correspond to homologs of *S. pombe* and *S. cerevisiae*, and are expected to be expressed in both. We note that the results are obtained in a blind fashion, for we do not separate *S. pombe* and *S. cerevisiae* probes in normalization. Therefore, the probe sets of *S. pombe* are used in both training and validation. Some differences among the three M-A plots are observed. This is not a surprise because the three genes *sch9*, *ras2*, *tor1* do play some different roles according to what we know.

We check the M-A plot of raw data without any normalization. That is, for three replicates of a mutant and the wild type, we summarize their expression values by the median polishing method. Then we calculate the average and difference expression of two strains and show the result by M-A plots, see Figure S2. The differentiation for probe sets of *S. pombe* is mainly distributed along the horizontal direction. This suggests that we can shift the differentiation of *S. cerevisiae* genes by the median of the differentiation of *S. pombe* genes. We compare the expressions resulted from this simple median-shift normalization with expression from the above method, the correlation coefficients are respectively 0.990, 0.970, 0.988 for *sch9*Δ, *ras2*Δ, *tor1*Δ.

The similarity indicates that quality of the microarrays is relatively good. Finally, the expression results are confirmed by quantitative RT-PCR for eleven genes and northern blots for two genes.

We calculated the one-sided Wilcoxon rank test score for each TIGO subset versus the rest genes, and ranked these subsets according to their corresponding q-values respectively for the up-regulated and down-regulated case. The same computation was carried out for KEGG pathways. These results are rather lengthy, and we report the most significant parts later in an integrative way. The complete spreadsheets can be found in the supplementary materials, Text S1, Text S3 and Text S4. The result of the cellular organelle analysis (GFP fusion localization) is summarized in Table 1, and the details can be found in Text S2.

**Table 1:** Positively- and negatively-regulated cellular organelles.

*Positively regulated cellular organelles*						
	sch9Δ/wt		ras2Δ/wt		tor1Δ/wt	
cellular organelle	p-value	q-value	p-value	q-value	p-value	q-value
ER	6.3E-06	3.8E-05	0.0	0.0	6.7E-16	9.4E-15
vacuole	1.2E-05	4.7E-05	0.0	0.0	2.3E-11	1.6E-10
vacuolar membrane	2.5E-04	6.4E-04	1.3E-04	4.8E-04	2.3E-06	8.1E-06
actin	2.6E-04	6.4E-04	0.18	0.24	2.3E-04	5.5E-04
punctate composite	0.0062	0.011	0.11	0.18	8.9E-06	2.5E-05
cytoplasm	9.1E-13	1.1E-11	2.1E-05	1.1E-04	1.1E-09	5.0E-09
***Negatively regulated cellular organelles***						
mitochondrion	2.6E-34	5.0E-33	1.2E-11	9.7E-11	6.8E-27	1.2E-25
nucleus	3.6E-06	2.3E-05	1.7E-23	2.7E-22	6.2E-12	3.7E-11
nucleolus	2.9E-11	2.8E-10	7.4E-08	3.9E-07	2.0E-17	1.8E-16
nuclear periphery	0.37	0.82	0.015	0.031	0.060	0.15
bud neck	0.31	0.82	5.3E-05	1.7E-04	0.017	0.051
spindle pole	0.0055	0.026	4.8E-05	1.7E-04	0.0053	0.019
bud	0.69	0.82	0.016	0.031	0.13	0.29
microtubule	0.010	0.040	0.0046	0.012	0.0020	0.0088

### Lower transcriptional activities

Our analysis shows that the overall transcription activities of the three long-lived mutants are relatively lower than those of the wild type. In fact, in the comparison of KEGG pathway activities, “basal transcription factors” and “RNA polymerase”, are among the most negatively regulated pathways, which also include “DNA polymerase”, “Cell cycle” and “Proteasome”, see Table 2. The basal transcription factors form a complex that acts as a general transcription machine. One explanation is that *sch9*Δ, *ras2*Δ, *tor1*Δ mutants in the nutrient-depleted environment can live a more economical life and hence a lower basal transcription is sufficient to maintain survival. In consistent with lower transcriptional level, proteasome, the complex in charge of protein degradation, is negatively regulated in the long-lived mutants. Moreover, in Table 3, we list the expression activities of relevant pre-transcription and post-transcription TIGO categories, which are all down-regulated with sufficient statistical evidence. From the perspective of protein localizations, we see in Table 1 that the expression activities of the compartment nucleus and nucleolus are significantly lower in the mutants *sch9*Δ, *ras2*Δ, *tor1*Δ compared to the wild type. It is noticed that reduction of transcriptional activities is most significant in *ras2*Δ, and this is confirmed by the overall expression profiles shown in Figure 3. All these evidences lead to the consensus that the three long-lived mutants somehow manage to lower their transcription activities in the SDC medium.

**Table 2:** Most negatively regulated KEGG pathways in the long-lived mutants compared to the wild type.

KEGG Pathway (down-regulated)	# genes	sch9Δ/wt		ras2Δ/wt		tor1Δ/wt	
		p-value	q-value	p-value	q-value	p-value	q-value
Basal transcription factors	23	3.2E-05	1.3E-03	2.1E-09	3.6E-07	1.6E-05	9.5E-04
RNA polymerase	29	0.0056	0.055	0.011	0.086	6.7E-03	0.060
DNA polymerase	9	0.017	0.117	5.8E-05	0.0017	0.0033	0.039
Aminoacyl-tRNA synthetases	37	0.0038	0.040	1.4E-04	2.7E-03	9.1E-05	2.0E-03
Cell cycle	105	0.0064	0.060	2.7E-12	9.4E-10	4.2E-05	0.0014
Proteasome	11	0.0037	0.040	9.2E-05	0.0020	0.0065	0.060

**Table 3:** Down-regulation of TIGO categories relating to transcription.

TIGO category (down-regulated)	# genes	sch9Δ/wt		ras2Δ/wt		tor1Δ/wt	
		p-value	q-value	p-value	q-value	p-value	q-value
general RNA polymerase II transcription factor activity	62	6.2-03	3.7E-02	2.7E-07	5.8E-06	7.3E-03	2.9E-02
DNA-directed RNA polymerase II, holoenzyme	74	2.0E-05	3.8E-04	8.6E-10	3.8E-08	1.7E-04	1.9E-03
transcription from RNA polymerase III promoter	38	9.8E-03	4.7E-02	5.8E-03	1.5E-02	9.5E-03	3.7E-02
nuclear mRNA splicing, via spliceosome	97	6.4E-04	7.1E-03	4.2E-09	1.4E-07	4.6E-04	3.7E-03
RNA splicing factor activity, transesterification mechanism	42	1.3E-02	5.9E-02	1.1E-04	8.2E-04	1.1E-02	3.9E-02
mRNA-nucleus export	60	3.2E-02	0.11	1.8E-04	1.2E-03	2.9E-04	2.7E-03
nuclear pore	50	3.3E-02	0.11	3.7E-04	2.1E-03	2.5E-03	1.3E-02

### Switch of energy pathway

The universal “currency” of chemical energy ATP in animal cells and most other non-photosynthetic cells is generated mainly by the aerobic oxidation process. In aerobic oxidation, glucose is metabolized to CO_2_ and H_2_O, and the released energy is converted to the chemical energy of phosphoanhydride bonds in ATP. The initial steps of oxidation of glucose, referred to as glycolysis, convert glucose into pyruvate. The reactions of glycolysis occur in the cytosol in both eukaryotes and prokaryotes and do not require O_2_. In contrast, the final steps of oxidation require O_2_ and occur in mitochondria in eukaryotes. The synthesis of ATP in mitochondria is driven by the flow of electrons from the reduced coenzymes NADH and FADH_2_ to O_2_. This oxidative phosphorylation process depends on the generation of proton-motive force across the inner membrane, with electron transport and proton pumping. Other than glycolysis, reactions in the mitochondria such as the citric acid cycle (TCA) also generate the reduced coenzymes NADH and FADH_2_.

**Figure 4:**
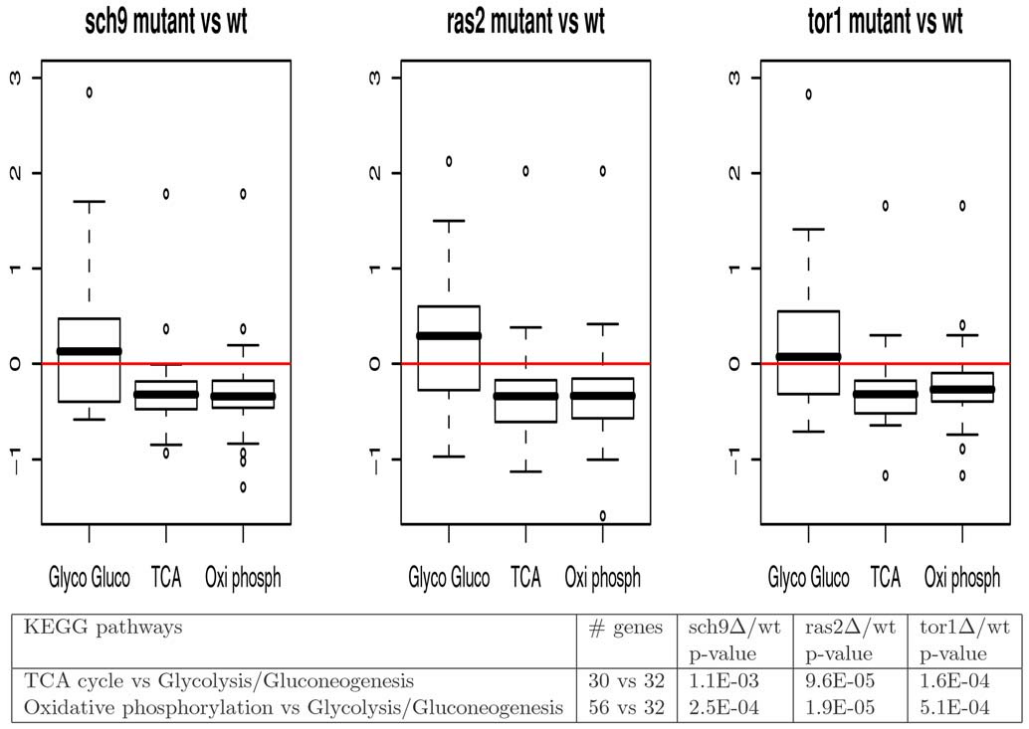
Comparison of Glycolysis/Gluconeogenesis, TCA, and Oxidative phosphorylation. Top panel: The box plots of the log-ratios of gene expressions for Glycolysis/Gluconeogenesis, TCA, and Oxidative phosphorylation in the long-lived mutants with respect to wild type; bottom: statistical significances of the above comparisons using the Wilcoxon rank test.

In our KEGG pathway analysis, the most up-regulated metabolic pathways common to all three mutants with respect to the wild type is Glycolysis/Gluconeogenesis. Since much is known about the aerobic oxidation process, we examine the details of the expression differentiation between the long-lived strains and wild type. Specifically, we compare the expression activities of the initial steps and final steps using the Wilcoxon rank test. At the bottom of Figure 4 we show the statistical results for the comparison of the KEGG pathways: glycolysis/gluconeogenesis versus TCA and oxidative phosphorylation. The analysis implies that in the long-lived mutants, TCA cycle and oxidative phosphorylation are negatively regulated compared with Glycolysis/Gluconeogenesis. The comparison is shown by box plots at the top of Figure 4. Usually yeast becomes hypo-metabolic (respiration rates decrease) around day 3–5. One explanation to the observation is that the long-lived become hypometabolic faster than the wild type.

*Hxt*2 and *Hxt*4 are both high-affinity glucose transporters, whose expressions are induced by low levels of glucose and repressed by high levels of glucose [27], [28]. Our result indicates that dysfunction of either *sch9*Δ, *ras2*Δ, or *tor1*Δ leads to significant up-regulation of *Hxt*2 (log-ratios 1.63, 0.83 1.54) and *Hxt*4 (log-ratios 1.15, 1.66, 0.67), and thereby a more efficient usage of glucose. Consistently, the TIGO category associated with monosaccharide catabolism is also positively modified. Genes in this TIGO category participate in chemical reactions that lead to breakdown of monosaccharides and polyhydric alcohols.

Significant changes of other related pathways are also observed. The fructose and mannose metabolism are positively regulated in *sch9*Δ, *ras2*Δ, and *tor1*Δ, the galactose metabolism, starch and sucrose metabolism are positively regulated in *ras2*Δ, although not as significant as the Glycolysis/Gluconeogenesis pathway. They indicate that the up-regulation of Glycolysis/Gluconeogenesis is associated with modifications of other catabolic pathways in the mutant cells.

### Change of compartment activity

In yeast, the reactions of TCA cycle, electron transport and oxidative phosphorylation occur inside mitochondria whereas those of glycolysis occur in cytosol. In aerobic conditions, oxidative phosphorylation is efficient to generate ATPs, but at the same time it produces the reactive oxygen species (ROS) as byproducts, which is thought to be one of the causes of ageing.

**Figure 5:**
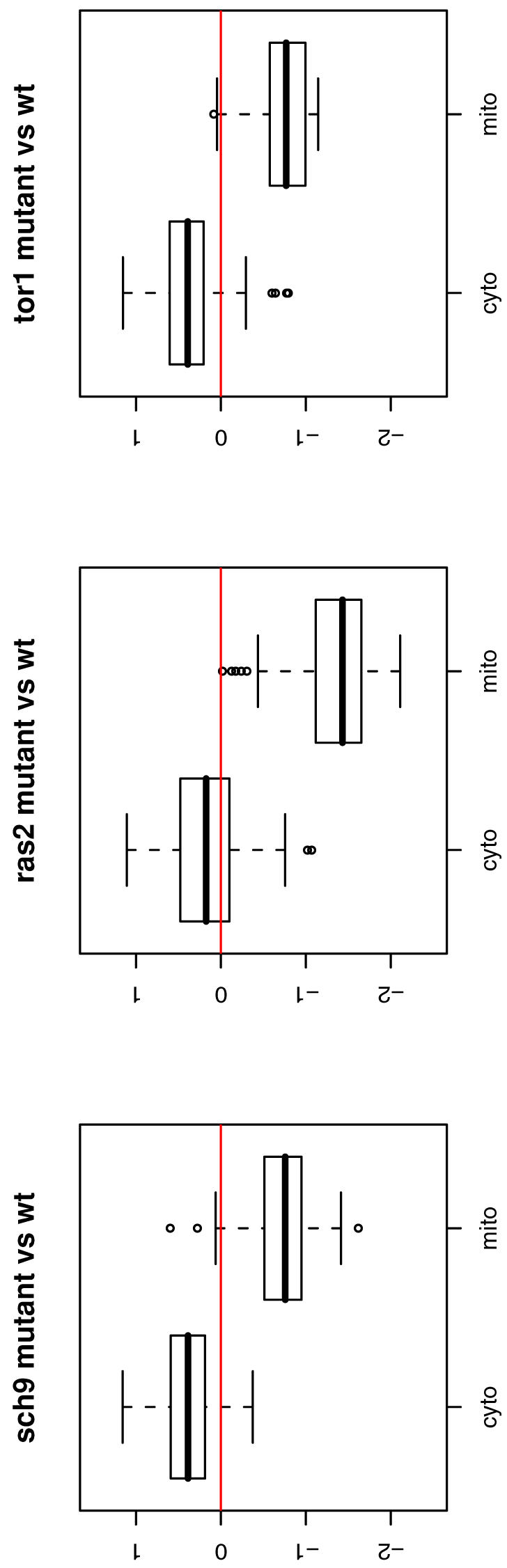
Box plots of fold changes between cytosolic and mitochondrial ribosome proteins. The box plots of expression log-ratios for the cytosolic and mitochondrial ribosome proteins in the long-lived mutants with respect to wild type. We also compare the expression differentiation by the Wilcoxon rank test and all three p-values are 0.00.

The change of energy pathways leads us to consider change of compartment activities. From Table 1, the overall expression levels of mitochondria is significantly lower in *sch9*Δ, *ras2*Δ, *tor1*Δ. Moreover, in Table 4 we examine the expression differentiations for all five TIGO categories specifically associated with mitochondria, which include mitochondrial large ribosomal subunit, mitochondrial small ribosomal subunit, mitochondrial inner membrane, mitochondrion organization and biogenesis and protein-mitochondrial targeting. Their expression activities are consistently down-regulated in all three mutants. Thus we hypothesize that the reduction of biological activities in mitochondria may lead to elongation of chronological life span in *S. cerevisiae*. In contrast, our GO analysis shows that the TIGO categories, cytosolic large ribosomal subunit (GO:0005842), and cytosolic small ribosomal subunit (GO:0005843) are positively regulated in all the long-lived mutants. Consistent with the GO analysis, the ribosome pathway excluding mitochondria ribosomal subunits is positively regulated in the KEGG analysis. Furthermore, we directly examine expression differentiation between cytosolic ribosomes and mitochondrial ribosomes by the Wilcoxon rank test and all p-values are less than 10^−10^. The comparison is illustrated by box plots in Figure 5.

**Table 4:** The transcription activities of the long-lived strains with respect to the wild type yeast for five TIGO categories that are associated with mitochondria.

Mitochondrial TIGO category (down-regulated)	# genes	sch9Δ/wt		ras2Δ/wt		tor1Δ/wt	
		p-value	q-value	p-value	q-value	p-value	q-value
mitochondrial large ribosomal subunit	43	4.3E-18	6.6E-16	1.5E-18	2.0E-16	1.6E-17	2.3E-15
mitochondrial small ribosomal subunit	34	1.9E-12	9.5E-11	1.8E-12	1.2E-10	7.5E-12	5.4E-10
mitochondrial inner membrane	158	7.1E-16	5.4E-14	6.7E-03	1.7E-02	5.4E-10	2.6E-08
Mitochondrion organization and biogenesis	95	6.5E-04	7.0E-03	1.4E-03	5.9E-03	6.5E-03	2.6E-02
protein-mitochondrial targeting	47	1.8E-05	3.8E-04	4.8E-02	8.6E-02	1.8E-04	1.9E-03

As shown in Table 1, ER-located and vacuole-located proteins are positively affected. The GO category (GO:0005789) endoplasmic reticulum membrane is up-regulated too. The endoplasmic reticulum is part of the endomembrane system, which modifies proteins, makes macromolecules, and transfers substances throughout the cell. In budding yeast cells, vacuoles are the storage compartments of amino acids and the detoxification compartments. Under conditions of starvation, proteins are degraded in vacuoles, which is called autophagy. The up-regulations of vacuole-located proteins may imply that autophagy in the cells of these long-lived mutants is enhanced to maintain survival in low nutrient conditions such as SDC medium.

### Differences among mutants

Despite the common expression patterns in *sch9*Δ, *ras2*Δ, and *tor1*Δ, we do observe various differences. r*as*2Δ shows the lowest overall transcriptional activities. In addition, we found those activities relating to mitosis in the mutant *ras2*Δ are all down-regulated, see Table 5. They include regulation of mitosis, mitotic sister chromatid segregation, and mitotic spindle organization and biogenesis. The activities in the cellular components (GO categories) such as spindle pole body, spindle pole body and microtubule cycle, bud neck, bud tip and incipient bud site, provide additional evidence. Similar and independent results are found in the cellular location analysis (Table 1). Other activities relating to DNA replication and DNA repair are down-regulated too. It is in consistency with the lower mitotic activities. It is known that Ras proteins regulate cell growth in response to nutrient availability through protein kinase A (PKA) activity, see [29] for references. Also Ras proteins have PKA-independent functions in mitosis and actin repolarization [30]. The expression profiles from our experiments provide systematic evidences to these stories. The down-regulation of the MAPK signaling pathway is significant in *ras*2Δ (q-value = 0.009), less slight in *tor1*Δ, and not in *sch9*Δ.

**Table 5:** The transcription activities of *ras2*Δ with respect to the wild type yeast for TIGO categories relating to mitosis.

TIGO category (down-regulated)	# genes	p-value	q-value
regulation of mitosis	48	5.1E-03	1.4E-02
mitotic sister chromatid segregation	55	5.6E-05	5.0E-04
mitotic spindle organization and biogenesis	42	1.3E-02	2.8E-02
spindle pole body	58	8.9E-03	2.1E-02
spindle pole body and microtubule cycle	37	5.5E-03	1.5E-02
condensed nuclear chromosome kinetochore	47	3.6E-05	3.4E-04
bud neck	108	1.1E-03	4.6E-03
bud tip	50	3.8E-04	2.1E-03
incipient bud site	35	9.5E-04	4.2E-03
invasive growth	30	9.5E-04	4.2E-03
DNA strand elongation	30	2.1E-03	7.2E-03
replication fork	39	4.9E-04	2.6E-03
chromatin remodeling complex	71	1.9E-08	5.0E-07
histone modification	59	1.2E-06	2.1E-05
double-strand break repair	41	7.0E-03	1.7E-02
nucleotide-excision repair	31	4.0E-04	2.2E-03

Some differences in metabolisms are also observed. For example, the inositol phosphate metabolism is down-regulated in *ras2*Δ, and *tor1*Δ (q-value = 0.013, 0.039), but not in *sch9*Δ. The phosphatidylinositol signaling system is down-regulated in *ras2*Δ, and *tor1*Δ (q-value = 0.036, 0.059), but not in *sch9*Δ. Although it is difficult to enumerate them all, differences among the three long-lived mutants, from another angle, suggest that the common expression patterns reported above are strongly linked to longevity.

## Discussion

To understand the mechanisms of ageing, we identified the common and characteristic differentiation in the transcriptional profiles of the three long-lived strains *sch9*Δ, *ras2*Δ, and *tor1*Δ. The success of our effort hinges on the measurement accuracy of mRNA expression levels. In the design of Affymetrix GeneChip®, multiple (11-20) 25-mer probes are used for each ORF (open reading frame) and they serve as within-block statistical replicates. In addition, we do observe higher probe specificity and other improvement in the recent yeast2.0 chips. It should be noted that cross-hybridization always exists and the measured values tend to be smaller than real differentiation.

Our analysis is based on the sub-array normalization that aims to improve accuracy and preserve differentiation. A simple linear function is sufficient, if not perfect, for the purpose of normalizing our yeast microarrays. First, all the microarray experiments were conducted under the same condition. Second, the estimated scale values are mostly in the range [0.7, 1.3]; see the histograms in Figure S1. It is argued in [23] that a simple linear function is sufficient in such a normalization case. Third, the hybridization is a complicated process with various uncontrollable factors. It is important to use a highly robust estimator of the linear function to eliminate unpredictable probe intensities. Least trimmed squares is an appropriate choice due to its robustness in several senses. Fourth, the fair modification of expression profiles from normalization should vary from one situation to another. In our case, by comparing Figure 3 and Figure S2, we feel the fair modification of expression profiles from normalization should not be large. We made an effort to preprocess this yeast microarray data set and examined the validity of the presented results from the perspectives of *S. pombe* probes sets, non-normalized expression profiles, and other considerations and supporting examples reported in our previous work. It is our belief that the future of functional genomics and proteomics lies not only in scale but also in measurement accuracy. To be consistent with the sub-array normalization, we use the median polishing summarization stratified by the reference selected from raw wild type arrays. Additional investigation of the reference effect is worthwhile in the future research.

The common changes of biological activities in the differentiation are inferred by integrating the expression profiles with biological subsets defined by cellular organelles, metabolism pathways, biological process, and molecular functions. We choose to use those instruments from three sources: cellular localization of proteins, KEGG, and Gene Ontology. Gene Ontology compiles results from literature along three dimensions, and some categories overlap with those from the other two sources. Similarly, we can make inference about the transcriptional regulation in long-lived mutants using expression profiles together with information of ChIP-chip and binding motifs. The results are described in other reports. We use the q-value method developed by Storey *et al*. [25], [26] to deal with the multiple test issue. The definitions and algorithms of q-values were initially obtained based on several assumptions, one of which is that the null distribution of the p-value is uniform[0,1]. In Figure S3, we show the histograms of p-values from the KEGG pathway analysis. Besides, the same genes could be shared by multiple subsets and dependence among hypotheses exists. The sensitivity of q-values to these assumptions in our study is a subtle problem and it is worth more investigation in our future work.

Other than subset-versus-all comparisons, we also make local comparisons between two directly-related subsets such as cytosolic and mitochondrial ribosome proteins. If the “directly-related gene subsets” are appropriately selected, in our opinion, the conclusion drawn from microarray studies can be greatly strengthened by this local inference approach. Our consensus inference is obtained by the cross-examination of the inferences drawn from different instruments.

Our results show that mutants *sch9*Δ, *ras2*Δ, and *tor1*Δ, which share the same phenotype: longer chronological life span, do share some common differentiation patterns. The commonality is particularly interesting in the presence of various differences. For example, the activities relating to mitosis in *ras2*Δ are significantly reduced. The significant and systematic expression differentiation underlying the phenotype is critical for understanding ageing in yeast. One such feature is lower pre- and post-transcriptional activities.

Another common characteristics in long-lived strains is the down-regulation of TCA cycle and oxidative phosphorylation. In contrast, the upstream of this process, the Glycolysis/Gluconeogenesis pathway, is slightly or moderately up-regulated. The up-regulation of genes relating to Glycolysis/Gluconeogenesis implies that mutant cells consume the carbon sources in a different manner compared to the wild type. The adaption may be achieved through a mechanism similar to that in CR. On the other hand, the down-regulation of genes relating to TCA cycle and oxidative phosphorylation indicates that mutant cells switch to alternative energy pathways that likely depend on glycolysis. Rea *et al.* [31] proposed a metabolic model to describe the “Energy switch” hypothesis for longevity mutants in *C. elegans*. They suggested that most, if not all, long-lived mutants in *C. elegans* utilize anaerobic mitochondrial fermentation, which do not involve the electron transport chain and generate fewer radical species. Our results indicate that the notion of “energy switch” may be relevant for explaining life span extension in *S. cerevisiae*. However, rather than anaerobic mitochondrial fermentation, in the yeast strains *sch9*Δ, *ras2*Δ, and *tor1*Δ, the alternative energy pathway is likely to involve glycolysis and occur in cytosol or organelles other than mitochondria.

Evidences from the analysis of cellular organelle, GO, and the comparison of cytosolic and mitochondrial ribosomes all indicate that activities of mitochondria are significantly reduced in *sch9*Δ, *ras2*Δ, and *tor1*Δ. In contrast, the expressions of cytosolic ribosomes are up-regulated. This change of compartment activities supports the ROS theory, which says reactive oxygen species (ROS) damage macromolecules and thereby accelerate ageing. The majority of cellular ROS (approximately 90%) is generated in mitochondria as a byproduct of oxidative phosphorylation during respiration [32]. A number of mutations affecting respiration have been found to increase life span, and at least some may achieve this by decreasing ROS levels [34]. According to our analysis, many of the down-regulated genes encode mitochondrial proteins; conversely, the expression levels of genes that encode proteins localized in mitochondria tend to be negatively regulated in the long-lived mutants. Particularly, in the long-lived mutants, TCA and oxidative phosphorylation are negatively affected, both of which occur in the mitochondria. As a consequence, respiration is reduced and thereby less ROS are produced. Our observations and implications are consistent with results from a systematic RNA interference (RNAi) screen of 5,690 *Caenorhabditis elegans* genes for gene inactivations that increase lifespan [34]. They found that genes important for mitochondrial function stand out as a principal group of genes affecting *C. elegans* lifespan. Our results in yeast suggest that reduction of mitochondrial activities closely relates to extension of the yeast chronological life span.

## Supporting Information

Figure S1 Histograms of the scale values in Figure 2.
(0.02 MB EPS)

Figure S2 M-A plots of data without normalization. In the M-A plots, the x, y coordinate value of a dot respectively show the average and difference of a gene expressions between the wild type and a mutant. Black: S. cerevisiae; Magenta: S. pombe. Top: sch9 mutant; Middle: ras2 mutant; Bottom: tor1 mutant. Two horizontal lines at ±0.15 are also plotted.
(0.86 MB EPS)

Figure S3 Histograms of the p-values in the KEGG Pathway analysis. p-values are calculated for one-sided Wilcoxon tests that compare differentiation between mutants and wild type on gene subsets defined by KEGG Pathways. Left: sch9 mutant vs. wild; Middle: ras2 mutant vs. wild type; Bottom: tor1 mutant vs. wild type.
(0.01 MB EPS)

Text S1 Spreadsheet of KEGG analysis
(0.05 MB XLS)

Text S2 Spreadsheet of Cellular organelle analysis
(0.00 MB XLS)

Text S3 Spreadsheet of TIGO analysis
(0.03 MB XLS)

Text S4 Information of the TIGO categories
(0.01 MB TXT)
